# Texture Analysis of Multiparametric Kidney MRI–A Non-Invasive Approach to Chronic Kidney Disease State

**DOI:** 10.3390/biomedicines14071575

**Published:** 2026-07-14

**Authors:** Marcin Majos, Artur Klepaczko, Katarzyna Szychowska, Ludomir Stefanczyk, Ilona Kurnatowska

**Affiliations:** 1 Department of Normal and Clinical Anatomy, Medical University of Lodz, 90-752 Lodz, Poland; 2 Medical Electronics Division, Institute of Electronics, Lodz University of Technology, 90-924 Lodz, Poland; 3 Department of Internal Diseases and Transplant Nephrology, Medical University of Lodz, 90-153 Lodz, Polandilona.kurnatowska@umed.lodz.pl (I.K.); 4 I Department of Radiology and Diagnostic Imaging, Medical University of Lodz, 90-153 Lodz, Poland

**Keywords:** magnetic resonance, T1-weighted images, T2-weighted images, DWI, multiparametric, chronic kidney disease

## Abstract

**Introduction**: The most common indication of chronic kidney disease (CKD) is a sudden deterioration of renal function. At that point, the only reliable test determining disease progression is the histopathological evaluation of material obtained during kidney biopsy. Although biopsy is currently the gold standard of diagnosis, the procedure is associated with a number of complications that further burden kidney function. The aim of this work is to evaluate the potential of multiparametric magnetic resonance image (MRI) in the assessment of the renal parenchyma state. **Material and Methods**: The study is based on kidney MRI examinations of healthy volunteers (group 1, n = 11) and patients suffering from CKD in the active phase (group 2, n = 29) or the non-active phase (group 3, n = 14), as confirmed by kidney biopsy. The T1-weighted images, T2-weighted images and DWI images were extracted and used to create algorithms, based on Support Vector Machine and Sequential Floating Forward Search, aimed to differentiate defined groups. **Results**: The final algorithms obtained the following metrics: for T1-weighted images, analysis–precision 91.5%, sensitivity 89.6%, specificity 94.8%; for T2-weighted images, analysis–precision 80.2%, sensitivity 79.0%, specificity 89.5%; for DWI images, analysis–precision 82.3%, sensitivity 82.1%, specificity 91.0%; and for multiparametric MRIs, analysis–precision 90.9%, sensitivity 90.4%, specificity 95.2%. **Conclusions**: Texture analysis of multiparametric MRI seems to have diagnostic potential in CKD and its development may reduce the frequency of invasive procedures such as kidney biopsy.

## 1. Introduction

One of the key challenges facing modern medicine today is the exponential growth in the incidence of chronic kidney disease (CKD), currently estimated to affect 10–13% of the population [1]. As CKD generally requires very costly treatment, such as hemodialysis and kidney transplantation, the disease places a significantly larger burden on health care systems compared to other common chronic diseases, such as hypertension or diabetes [2,3,4,5,6].

One therapeutic approach is based on the ongoing development of diagnostic methods enabling more personalized treatment [7]. Currently, the diagnosis and monitoring of CKD is largely based on an extensive panel of laboratory tests, the core of which comprises the assessment of blood creatinine and urea levels and proteinuria [8]. Other diagnostic methods are much less frequently used and are typically reserved for exceptional situations.

The most common indication of CKD is a sudden deterioration in the renal function observed in laboratory analysis [9]; such changes are characteristic of an exacerbation to an active phase. At that point, the only reliable test determining disease evolution is the histopathological evaluation of material obtained during kidney biopsy [10]. Kidney biopsy is recommended by KDIGO [11] to determine CKD ethology in adult patients due to its informative potential and relatively low complication rates for invasive procedures. In pediatric populations, it is recommended to perform genetic examination due to the high possibility of hereditary CKD.

The analysis of kidney MRI images is a matter of growing interest in CKD diagnosis [12]. For example, in a study based on T2-wighted images from 1299 patients from five countries, Karatepe et al [13] employed an AI-based algorithm to distinguish CKD patients from healthy controls with 98.31% precision. Elsewhere, an algorithm based on the texture analysis of T2-weighted images was used to detect developing CKD in 108 patients with bipolar disorder who underwent lithium treatment [14]. The algorithm was characterized by an AUC of 0.85.

The texture analysis of a single MRI sequence has been found to have considerable potential in determining the renal status in patients with CKD [15]. The aim of this work is to evaluate the potential of multiparametric magnetic resonance image (MRI) in the assessment of the renal parenchyma; it examines whether the combination of T1, T2, and DWI texture features increases the classification accuracy compared to algorithms based only on one of these sequences.

## 2. Materials and Methods

Two groups of participants were included in the study. The first consisted of healthy volunteers with no history of kidney disease who underwent multiparametric renal MRI. These were allocated to Group 1. The second group comprised patients of the Nephrology Department of our hospital, recruited consecutively from November 2020 to January 2022; participants from second group underwent renal biopsy due to suspected exacerbation of CKD and received multiparametric MRI 24 hours after the biopsy. Based on the histopathology results, the second group was divided into two subgroups: those with active visible histopathological inflammatory infiltration signs (Group 2) and those with inactive changes with absent or minimal inflammatory infiltration or histopathological features of advanced kidney fibrosis (Group 3).

**Figure 1:**
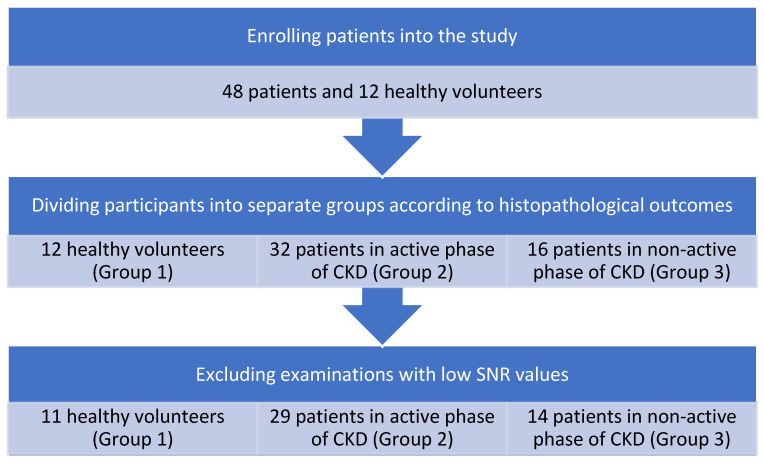
Flowchart of patient inclusion and divisions in the study.

Six participants were excluded due to low SNR values. A flowchart of the process is presented in Figure 1.

Therefore, 11 patients belonging to Group 1, 29 belonging to Group 2 and 14 belonging to Group 3 were included in the study. Their data are presented in Table 1.

The following etiologies were identified for CKD among the patients: focal segmental glomerular sclerosis (Group 2—nine patients, Group 3—four patients), vasculitis (Group 2—four patients, Group 3—zero patients), lupus nephritis (Group 2—five patients, Group 3—one patient), tubulointerstitial nephritis (Group 2—two patients, Group 3—two patients), IgA nephropathy (Group 2—four patients, Group 3—two patients), membranous nephropathy (Group 2—five patients, Group 3—zero patients), diabetes-related nephropathy (Group 2—zero patients, Group 3—one patient); four patients had end-stage kidney disease. Patients in Group 1 did not present comorbidities. For patients in Group 2, 9 were treated for hypertension and 4 for dyslipidemia. For patients from Group 3, 8 were treated for hypertension, 2 for dyslipidemia and 4 for diabetes.

All MRI examinations were performed on a 3T Magnetom Vida unit (Siemens Healthcare GmbH, Erlangen, Germany) 24 h after renal biopsy. The MRI study protocol consisted of a sequence of dixon-dependent T1-weighted images (TR = 4 ms, TE1 = 1.26 ms, TE2 = 2.4 ms, TA = 14.78 ms) and T2-weighted HASTE images with fat saturation (TR= 1350 ms, TE = 80 ms, TA = 0.51 ms) and ADC map (TR = 1350 ms, TE = 80 ms, TA = 0.51 ms).

In the healthy volunteer group, the analysis included images of both kidneys as they did not receive a kidney biopsy. However, for the CKD patients, images of the biopsied kidney were not used to exclude the influence of post-biopsy changes.

The trial was approved by the Bioethical Committee associated with the Medical University of Lodz (decision RNN/206/20/KE, dated 8 September 2020).

As relatively few subjects were included, it was not possible to use modern deep learning technology to develop classification models. Therefore, a classic approach was taken based on texture feature extraction, selection and machine learning, as described below.

**Figure 2:**
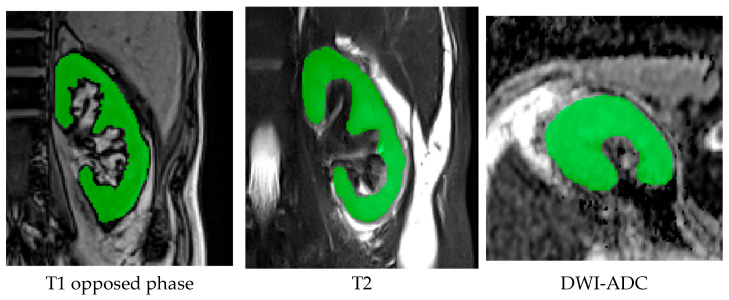
Coronal (T1 and T2) and axial (DWI) cross sections selected for analysis with manually delineated regions of interest (ROI is delineated with green color). Image is selected from a healthy volunteer (Group 1, eGFR > 60 mL/min./1.73 m^2^).

The data processing pipeline consisted of the following key stages:

Texture Feature Extraction. A comprehensive set of a total of 221 texture features was extracted from the images, including the image histogram, an autoregressive model, image gradient statistics, run-length matrix features, and co-occurrence matrix features. All features were calculated using qMaZda software (qMazda, version 2017, Lodz, Poland) [16,17] within regions of interest (ROI) manually delineated in the respective images (see Figure 2). For each CKD patient, only one such ROI was manually delineated in a given image modality (T1, T2, DWI), resulting in three ROIs per subject. For each control subject, six ROIs were constructed, as in this group, both kidneys were included in the analysis. All ROIs were delineated by an expert with over 10 years of experience in renal MR image processing who was blinded to clinical group assignment. ROI placement followed a standardized protocol to minimize operator-dependent variability.

Feature Standardization. All extracted features were standardized to ensure a sample mean of 0 and a standard deviation of 1, mitigating the influence of differing scales on the subsequent analysis.

Dataset Oversampling. To address class imbalance and increase the robustness of the model, the dataset was oversampled 20 times. Each oversampling iteration was performed with a different random seed for the number generator, creating 20 distinct variations of the augmented dataset. Oversampling was performed using the SMOTE–Tomek technique, which combines Synthetic Minority Oversampling Technique (SMOTE) with so-called Tomek link removal. SMOTE creates synthetic minority-class samples by interpolating between existing data points, effectively increasing minority-class representation. Tomek links are then identified and removed to eliminate borderline or overlapping samples, helping to clean class boundaries. Together, SMOTE–Tomek both balances the dataset and reduces noise, improving the quality of the training data. While this method improves class balance and boundary clarity, it may also introduce biases due to the artificial nature of the synthetic samples and the modified class distributions. To reduce the variance associated with synthetic augmentation, the procedure was repeated 20 times with different random seeds, producing 20 independently balanced datasets for feature selection. Because the minority classes were substantially underrepresented, classification models trained on the original dataset tended to favor the majority class and produced unstable estimates of feature importance. Repeated SMOTE–Tomek oversampling was therefore used to improve class balance and to identify features that remained consistently relevant across multiple independently augmented versions of the dataset.

Significant Feature Selection. Given that the number of extracted features exceeds the sample size by more than a four-to-one ratio, we implemented a robust multi-stage feature selection protocol specifically designed to minimize overfitting by combining dimensionality reduction, cross-validated subset evaluation, and stability analysis across multiple independently oversampled datasets.

For each of the 20 oversampled datasets, feature selection was performed independently using only the data available within that dataset before classifier evaluation:

○ Initial Single-Feature Screening. The 10 best individual features, which minimized the error of a Support Vector Machine (SVM) classifier, were selected to form an initial subset for the subsequent step. Here, as well as in all subsequent steps involving SVM training, we used the radial basis function as a non-linear kernel mapping parameterized with a gamma value equal to the reciprocal of the data dimension.
○ Subset Selection via SFFS. The Sequential Floating Forward Search (SFFS) algorithm was applied to this initial subset to identify a significant feature subset. The wrapped classifier model was again the SVM, whose performance for a given feature subset was evaluated as the classification accuracy estimate in the 5-fold cross-validation mode.

The final set of features was determined based on their frequency of selection across all 20 experiments. For each feature, a selection frequency (the number of times it was chosen by the SFFS algorithm across all datasets) was calculated. A threshold frequency, corresponding to the 99th percentile of these counts, was established. Features whose selection frequency was equal to or greater than this threshold were retained for the final model. The 99th-percentile threshold for selection frequency was chosen to ensure that only the most stable and consistently selected features were retained across all 20 independently oversampled datasets. Lower thresholds were tested in preliminary analyses, but they resulted in substantially larger and less stable feature sets, increasing the risk of overfitting. The 99th-percentile cutoff therefore represented a balance between model simplicity and robustness. The final feature set comprised between 10 and 12 features, depending on the imaging modality, as detailed in Table 2.

The entire data processing stage (feature standardization, oversampling, feature selection) was accomplished using methods implemented in Weka software (Weka 3.8) [18]. The definitions of the classification evaluation metrics are presented in Table 3.

Data Classification. Using the feature subsets selected independently within each oversampled dataset, we conducted a series of classification experiments using the same SVM framework. Again, these experiments were performed using the SVM algorithm with RBF kernel and data oversampling. The RBF’s γ parameter was set to 1/*d* (*d* = number of features), following common practice in texture-based classification, while the *C* parameter was kept at the Weka default setting (*C* = 1) to maintain comparability across experiments and avoid overfitting through excessive hyperparameter tuning.

The oversampling technique was adopted to robustly estimate the classifier performance and ensure generalizability in the context of a limited dataset. A small sample size inherently increases the risk of model overfitting and yields performance metrics with high variance, making it difficult to draw statistically sound conclusions. To mitigate this, the oversampling procedure was integrated into a repeated random sub-sampling framework, analogous to bootstrapping principles. By executing the experiment multiple times (100 iterations in the case of classification stage), each with a unique random seed governing the oversampling, we effectively generated multiple varied versions of the training data. This approach allows for a more reliable estimation of the true error distribution of the classifier. The aggregation of the results across all iterations enables the calculation of confidence intervals for the performance metrics, thereby providing a measure of their stability and precision. Consequently, this methodology strengthens the statistical credibility of the findings, ensuring that the evaluated performance is not an artifact of a single fortunate random split of the data but a consistent property of the proposed model.

To evaluate the performance of the classification within each feature domain, as well as for the features combined from all imaging methods, the following evaluation metrics were computed:

- Classification accuracy averaged (ACC_20_) over 20 oversampled data sets, each described by a unique feature subset selected specifically for a given sample.
- The mean sensitivity or true positive rate (TPR), specificity or true negative rate (TNR), false positive rate (FPR), precision, and F1-Measure were computed across 100 oversampled datasets, all sharing a consistent final feature set derived from their frequency of selection. Each metric was calculated separately for every class and then averaged. The formal definitions of all evaluation metrics are provided in Table 3.

The final classifier evaluation across 100 iterations was conducted using only the features retained through selection-frequency analysis, ensuring that the data used for feature selection were not reused for performance estimation within the same iteration.

Imaging modalities. The above-described procedures were repeated for three distinct imaging methods implemented in the study, i.e., T1-, T2-, and diffusion-weighted images, as well as their combination. Hence, four subsets of features we extracted, selected and evaluated: three for separate imaging and one for their common cross-modal representation. The key outcome of this analysis is to create a multiparametric multi-class algorithm classifying different stages of CKD.

## 3. Results

The texture features selected for each imaging method are presented in Table 2. It is important to note that the number of finally selected features may differ depending on the percentile threshold value, which was separately determined in each case. Where necessary, the original feature names assigned in qMaZda software are preceded by prefixes indicating the source image for a given descriptor. Hence, T1IP stands for in-phase T1-weighted image in Dixon-Vibe method, T1OP—opposed phase image, ADC—apparent diffusion coefficient map from DWI imaging, and T2 is simply a T2-weighted image. Specific feature names are given previously [16] and in the qMaZda documentation [19]. Most importantly, ‘Glcm’ indicates grey-level co-occurrence matrix, ‘Glrm’—grey-level run-length matrix, and ‘Hist’—histogram-based features. The letter directly following the texture extraction shortcut reflects the direction of the pixel neighborhoods in which a feature is calculated (H—horizontal, V—vertical, Z—45-degree inclination, and N—135-degree inclination). In the case of ‘Glcm’, the consecutive digit (1–3) denotes the offset of the pixel neighborhood.

The precise final metrics of each created algorithm are presented in Table 4, Table 5, Table 6 and Table 7 with a multiclass classification algorithm presented in Table 7: this is the main result of the analysis.

## 4. Discussion

The present study is the first to use and evaluate the potential of histopathological analysis based on multiparametric kidney MRI images to determine the state of the kidney during possible CKD exacerbation.

Our findings indicate that the best differentiation between subject categories was offered by a combination of all feature types of all accessible sequences. However, the classification performance differs between image types, with the T1-weighted imaging significantly surpassing the other two sequences. Similarly, previous studies report T1-weighted images to have high specificity in detecting kidney fibrosis and edema on the microscopic level, whereas T2-weighted images and DWI offer better performance in estimating renal perfusion and oxygenation levels [20,21,22]. Hence, our findings suggest that pathological mechanisms influencing T1 images, i.e., microscopic fibrosis and edema, have the largest influence on CKD imaging at the histopathological level.

In addition, the selected features used by our algorithms correspond to varied pixel neighborhood directions, indicating that the tissue changes affecting specific texture alterations do not have any dominant direction. Hence, MRI may have an ability to detect the destruction of the well-organized microscopic kidney structures occurring during the development of CKD, as described in histopathological trials [23,24,25,26,27].

The earliest studies on the use of MRI for renal diagnostics found the T1-dependent signals of the renal cortex and medulla to vary with the increased oxygenation of circulating blood and dehydration, with the cortex values varying more widely than those in the medulla [28,29]. As a result, basic T1- and T2-dependent MRI sequences were rapidly incorporated into diagnostic protocols for renal pathology. Some researchers have also proposed the use of the corticomedullary differentiation ratio as an objective and more sensitive biomarker of renal pathology [30,31]. Subsequent studies suggest that the approach may be useful for assessing the development of specific renal pathologies, particularly the use of T1-weighted images in the evaluation of transplanted kidneys [32]. In recent studies, the obtained MRI data were found to provide a clear picture of the development of acute tubular necrosis and of acute and chronic rejection of the transplanted kidney [20,33]. All these reports indirectly confirm the method to be suitable for monitoring renal status.

Unfortunately, to date, only three studies have addressed the value of T1-weighted image textures in evaluating CKD. Of these, two examine the relationships between texture features and organ function as represented by GFR values and their ability to distinguish healthy patients from those suffering from CKD in the course of diabetes [34,35]. While their findings indirectly support those of the present study, both studies lack histopathological validation, and all of the patients are presumably in a non-active phase of CKD.

Few studies have evaluated the use of isolated T2-weighted image analysis in determining kidney status. Nevertheless, some findings suggest that T2 signal values may reflect patient hydration. T2-weighted images also appear to be particularly effective in detecting areas of hypoxia resulting from renal ischemia and areas of inflammatory and edematous changes [36,37]. Importantly, all of these phenomena play important roles in the course of CKD [21,22].

Although renal T2 signal values alone do not provide as clear a picture as T1-weighted imaging, several studies have explored their value in texture analysis. Three studies found the texture features of renal MRI examinations to correlate with the GFR values and to accurately stratify patients according to CKD development [35,38,39]. However, these studies did not examine the relationship between texture analysis and histopathological image, but they compared radiological features with stratified images of numerous native kidneys [35,39] and with limited numbers of renal grafts [38].

Our present study presents a more efficient algorithm than previous research using T2-weighted images alone; our proposed approach is also completely automated and based solely on texture features, without requiring additional morphological data of the kidney.

While no studies have so far evaluated the texture of diffusion maps in isolation, analyses of DWI signal intensity in CKD have found such sequences to play a potentially effective role in renal diagnosis. Two studies report that retrograde changes in CKD correlate with values from diffusion maps, which indirectly confirms our present findings [40,41]. Several publications indicate that the intensity of DWI signals can be used to distinguish healthy volunteers from those with CKD [42,43,44,45,46]. Other reports suggest that the DWI signal intensity may be indicative of renal function by serving as an estimate of the GFR [47,48,49]. All these reports confirm our present observations regarding the changes in diffusion occurring in the kidneys during CKD; however, these associations are indirect, as they focus on stratifying patients based on GFR values and only include those in the non-active phase of CKD.

A similar texture analysis protocol based on all the MRI sequences used in the present study has been described in a previous study [34]. Its algorithm used for grouping patients according to their GFR values was found to have very good sensitivity and specificity. While the manuscript does not include any direct reference to renal histopathology, their results confirm that texture analysis is a valuable tool in the diagnosis of CKD.

The most common histopathologically based studies of radiomics in CKD are those concentrating on kidney fibrosis. Wang et al. [50] evaluate multiparametric MRI consisting of IVIM, ASL, phase-contrast MRI, T1 mapping, and BOLD sequences as a modality for determining the severity of renal fibrosis. Their algorithm, trained on images of 116 MRI scans, was able to divide their patients into groups with mid and severe renal fibrosis with a sensitivity of 0.91 and a specificity of 0.73. However, while their study confirms the potential of MRI to detect the stage of renal fibrosis, it did not include a control group of healthy subjects; as such, it is impossible to confirm whether it can distinguish CKD patients from those not affected by CKD. A similar study by Zha et al. [51] also failed to include a group of healthy controls. Nevertheless, their algorithm achieved good AUC matrices, i.e. 0.861; more importantly, it was trained and tested on datasets from two centers. Hence, MRI image analysis may have the potential to become an effective diagnostic method that is independent of MRI vendor and scanning protocol.

Kidney biopsy has a firmly-established place in the diagnostic algorithm, and is unlikely to be replaced by MRI evaluation as a diagnostic method for CKD [11]. Despite this, texture analysis may be used to avoid multiple biopsies of the same patient. If a patient with a previous histopathological diagnosis, with a confirmed ethology of CKD, arrives at the Nephrology Department, a fast image analysis using an MRI scan would clearly be preferable to conducting another kidney biopsy. However, our algorithm, despite having promising metrics, is not yet ready for such application: further large-scale multicenter trials are required. Even though texture analysis should not be dependent on the MRI scanner manufacturer or the precise scanning algorithm, external validation is absolutely essential.

Image analysis related to CKD is not restricted to T1-weighted, T2-weighted images or DWI. Many MRI studies have evaluated advanced functional MRI sequences such as ALS, intravoxel incoherent motion, BOLD or phase-contrast MRI [52,53,54,55,56]. In addition, multiparametric ultrasound [57,58] and computed tomography [59], among others, can be used in the diagnosis and monitoring of CKD.

Despite this, the present study evaluates three primary MRI sequences for several reasons. Most importantly, it is desirable to keep the protocol short, as patients in the acute phase of CKD are not able to lay still for too long. Secondly, a short protocol is more widely applicable: in our area, many MRI systems do not include abdominal ALS or BOLD sequences or may not include official diagnostic stations.

The study has some limitations. Firstly, due to the very specific nature of the recruitment process, the study only included a relatively small number of participants, particularly regarding the healthy controls. This limitation is typical for studies requiring histopathological verification, but it inevitably reduces the statistical power and limits the generalizability. As our conclusions were made on a basis of such a limited study group, they need further validation on larger multi-center cohorts. Moreover, due to the nature of the disease and the rate of progression of the acute phase, it was not possible to prepare the patients for analysis, for example, by influencing their hydration level. In addition, small datasets increase the risk of model overfitting, despite the use of repeated experiments, confidence-interval reporting, and oversampling. Therefore, while the results demonstrate promising diagnostic performance, they should be considered as preliminary.

In addition, the present study was not designed to evaluate clinical decision-making utility. Analyses such as calibration assessment and decision curve analysis would require externally-validated probability estimates and clinically-defined decision thresholds. These evaluations should therefore be considered in future multi-center studies. Furthermore, although oversampling was used to address class imbalance, synthetic data cannot fully replicate the heterogeneity of real biological variability. This may influence the representation of minority groups and introduce subtle biases in feature distributions. Lastly, because ROI segmentation was manual and not repeated by an independent observer, inter-observer variability cannot be quantified and remains another methodological limitation.

Nevertheless, despite these limitations, the study presents an efficient approach to the use of machine learning in the interpretation of kidney texture features in CKD patients of various ethology. Importantly, the applied methodological procedures, reinforced by the reproducible performance observed over 100 oversampled variants of the dataset, suggest that the reported results are reliable and not artifacts introduced by small sample size or class imbalance.

## 5. Conclusions

Texture analysis of multiparametric MRI seems to have diagnostic potential in chronic kidney disease, and its development may reduce the frequency of invasive procedures such as kidney biopsy.

## Figures and Tables

**Table 1:** Presentation of patients’ basic characteristics. Group 1—healthy volunteers, Group 2—patients with active phase of CKD, Group 3—patients with non-active phase of CKD. SD—standard deviation.

Group	n	Sex		Mean Age (Years)	Mean eGFR (ml/min./1.73 m^2^)
		Female	Male		
1	11	7	4	43.22 (SD = 9.55)	60<
2	29	16	13	50.46 (SD = 16.54)	42.9 (SD = 18.59)
3	14	6	8	56.16 (SD = 15.14)	26.6 (SD = 15.21)

**Table 2:** Selected texture features used to create algorithms taken from each sequence.

T1-Weighted	T2-Weighted	DWI	All Modalities
T1IP_GlcmN1SumAverg T1OP_HistPerc99 T1OP_GlcmH2SumAverg T1OP_GlcmH3SumAverg T1OP_GlcmV2SumAverg T1OP_GlcmZ1ClustPrm T1OP_GlcmZ2SumAverg T1OP_GlcmN2SumAverg T1OP_YS5GlcmN3ClustPrm	GrlmVRLNonUni GlcmH1ClustPrm GlcmH3ClustPrm GlcmV2ClustShd GlcmV3ClustPrm GlcmZ1SumAverg GlcmZ1ClustShd GlcmZ2ClustPrm GlcmN2ClustPrm GlcmN3ClustPrm	HistMaxm01 HistDomn01 GrlmVGLevNonUn GrlmZLngREmph GrlmZShrtREmp GrlmZFraction GrlmZMRLNonUni GrlmNGLevNonUn GlcmH2ClustPrm GlcmZ1InvDfMom	ADC_GrlmZShrtREmp ADC_GrlmZMRLNonUni ADC_GlcmH3ClustPrm ADC_GlcmV2DifVarnc T1OP_HistMaxm01 T1OP_GlcmV2SumAverg T1OP_GlcmZ1ClustPrm T1OP_GlcmN3ClustPrm T2_HistPerc10 T2_GlcmV1SumAverg T2_GlcmV2SumVarnc T2_GlcmV3SumVarnc

**Table 3:** Definitions of classification evaluation metrics.

Metric	Formula	Description
ACC	ACC = (Σ TP_i_)/N	Overall proportion of correctly classified samples across all classes; ΣTP_i_ sums true positives over all classes, and *N* is the total number of samples.
Sensitivity (True Positive Rate, TPR)	Sensitivity = TP/(TP + FN)	Proportion of actual positives correctly identified by the model.
Specificity (True Negative Rate, TNR)	Specificity = TN/(TN + FP)	Proportion of actual negatives correctly identified by the classifier.
False Positive Rate (FPR)	FPR = FP/(FP + TN)	Proportion of actual negatives incorrectly classified as positives.
Precision	Precision = TP/(TP + FP)	Fraction of predicted positives that are true positives.
F1-Measure	F1 = 2 × (Precision × TPR)/(Precision + TPR)	Harmonic mean of precision and recall (TPR), balancing both.

**Table 4:** Precise metrics characterizing algorithm based on T1-weighted features.

	Mean	95% Confidence Interval
ACC_20_	0.9624	(0.9518; 0.9729)
Sensitivity (TPR, Recall)	0.8956	(0.8814; 0.9097)
Specificity (TNR)	0.9478	(0.9407; 0.9549)
False positive rate	0.0522	(0.0451; 0.0593)
Precision	0.9152	(0.9046; 0.9258)
F1	0.8968	(0.8828; 0.9109)

**Table 5:** Precise metrics characterizing algorithm based on T2-weighted features.

	Mean	95% Confidence Interval
ACC_20_	0.9103	(0.8833; 0.9373)
Sensitivity (TPR, Recall)	0.7900	(0.7693; 0.8107)
Specificity (TNR)	0.8950	(0.8847; 0.9053)
False positive rate	0.1050	(0.0947; 0.1153)
Precision	0.8021	(0.7831; 0.8212)
F1	0.7891	(0.7682; 0.8010)

**Table 6:** Precise metrics characterizing algorithm based on DWI-weighted features.

	Mean	95% Confidence Interval
ACC_20_	0.9144	(0.8976; 0.9312)
Sensitivity (TPR, Recall)	0.8206	(0.7962; 0.8449)
Specificity (TNR)	0.9103	(0.8981; 0.9225)
False positive rate	0.0897	(0.0775; 0.1019)
Precision	0.8229	(0.7978; 0.8479)
F1	0.8173	(0.7918; 0.8427)

**Table 7:** Overall metrics of multiparametric algorithm.

	Mean	95% Confidence Interval
ACC_20_	0.9784	(0.9678; 0.9890)
Sensitivity (TPR, Recall)	0.9044	(0.8909; 0.9181)
Specificity (TNR)	0.9522	(0.9454; 0.9590)
False positive rate	0.0478	(0.0410; 0.0546)
Precision	0.9086	(0.8958; 0.9214)
F1	0.9045	(0.8909; 0.9181)

## Data Availability

The raw data supporting the conclusions of this article will be made available by the authors on request.
